# Implication of New WHO Growth Standards on Identification of Risk Factors and Estimated Prevalence of Malnutrition in Rural Malawian Infants

**DOI:** 10.1371/journal.pone.0002684

**Published:** 2008-07-16

**Authors:** Marc-André Prost, Andreas Jahn, Sian Floyd, Hazzie Mvula, Eleneus Mwaiyeghele, Venance Mwinuka, Thomas Mhango, Amelia C. Crampin, Nuala McGrath, Paul E. M. Fine, Judith R. Glynn

**Affiliations:** 1 Epidemiology and Population Health Department, London School of Hygiene & Tropical Medicine, London, United Kingdom; 2 Karonga Prevention Study, Chilumba, Malawi; University of Cape Town, South Africa

## Abstract

**Background:**

The World Health Organization (WHO) released new Child Growth Standards in 2006 to replace the current National Center for Health Statistics (NCHS) growth reference. We assessed how switching from the NCHS to the newly released WHO Growth Standards affects the estimated prevalence of wasting, underweight and stunting, and the pattern of risk factors identified.

**Methodology/Principal Findings:**

Data were drawn from a village-informant driven Demographic Surveillance System in Northern Malawi. Children (n = 1328) were visited twice at 0–4 months and 11–15 months. Data were collected on the demographic and socio-economic environment of the child, health history, maternal and child anthropometry and child feeding practices. Weight-for-length, weight-for-age and length-for-age were derived in z-scores using the two growth references. In early infancy, prevalence estimates were 2.9, 6.1, and 8.5 fold higher for stunting, underweight, and wasting respectively using the WHO standards compared to NCHS reference (p<0.001 for all). At one year, prevalence estimates for wasting and stunting did not differ significantly according to reference used, but the prevalence of underweight was half that with the NCHS reference (p<0.001). Patterns of risk factors were similar with the two growth references for all outcomes at one year although the strength of association was higher with WHO standards.

**Conclusions/Significance:**

Differences in prevalence estimates differed in magnitude but not direction from previous studies. The scale of these differences depends on the population's nutritional status thus it should not be assumed a priori. The increase in estimated prevalence of wasting in early infancy has implications for feeding programs targeting lactating mothers and ante-natal multiple micronutrients supplementation to tackle small birth size. Risk factors identified using WHO standards remain comparable with findings based on the NCHS reference in similar settings. Further research should aim to identify whether the young infants additionally diagnosed as malnourished by this new standard are more appropriate targets for interventions than those identified with the NCHS reference.

## Introduction

In 2006 the World Health Organization (WHO) introduced new Child Growth Standards (the WHO standards) aimed at replacing the US National Center for Health Statistic (NCHS) growth reference (the NCHS reference) [1]. The NCHS reference had been criticized for its lack of generalizability stemming from the ethnic homogeneity of the sample used, and the fact that individuals were predominantly bottle-fed. In addition, inadequate measurement frequencies during this period of rapid growth have resulted in imprecise characterization of early infancy growth trajectories [2], [3]. The WHO standards aim to represent how children should grow rather then how they actually grow. They are based on an international multicenter exclusively breast-fed sample of healthy children living in the most favorable conditions to achieve their full genetic growth potential [1].

Despite criticism, since its introduction in 1979 the NCHS reference has been adopted by national programs in more than one hundred countries for growth monitoring purposes and by major Non-Governmental Organizations (NGO) intervening in the field of nutrition in less developed countries [4]. It has proved to be reliable for identifying children at increased risk of dying in a variety of contexts. The wide acceptance and use of this reference has allowed international comparisons and time-trends analyses. Adopting a new growth standard may impair our ability to perform such comparisons.

The WHO standards were tested in 4 countries prior to being formally recommended [5]. The classification of children's growth based on length/height-for-age and weight-for-age was tested against standardized clinical assessments. The authors found good agreement between the two methods and concluded on the technical soundness of the standard. However comparison between the WHO standards and both the NCHS reference and the Center for Disease Control and Prevention 2000 Growth Chart highlighted important discrepancies in the estimation of the prevalence of malnutrition, particularly marked in infancy and likely due to samples characteristics [6], [7]. The estimated prevalence of wasting (weight-for-height/length <-2 z-score), underweight (weight-for-age <-2 z-score) and stunting (height/length-for-age <-2 z-score) were considerably greater in the first 5 months of life when using the WHO standards than with the NCHS reference in a secondary analysis of longitudinal data from Bangladesh and the Dominican Republic [7]. Similarly, secondary analyses of anthropometric data from refugees aged 6–59 months from Kenya, Algeria and Bangladesh found a significantly higher prevalence of severe wasting (<-3 z-score) but not total wasting (<-2 z-score) with the WHO standards than with the NCHS reference [8]. The implications of using the WHO standards on the measured prevalence of these nutritional disorders are not yet fully understood but are likely to lead to increased estimates of malnutrition, particularly in infancy. The consequences of this increase on the identification of early risk factors for weight and height faltering have not been explored.

The 2004 Demographic and Health Survey (DHS) showed that the nutritional status of the under-5 population in Malawi is fairly typical of that of a sub-Saharan African country, with Malawi being in the middle range for the prevalence of wasting and underweight and the top range for stunting among 19 other countries [9], [10]. Despite a marked decrease in the 1970's–80's, trends in the prevalence of underweight and stunting have been plateauing if not increasing in recent years [11], [12]. As of 2006, the national prevalence of childhood underweight, wasting and stunting calculated using the NCHS reference stood at 22%, 5% and 48% respectively [12]. To help understand how adopting the new WHO standards may influence the estimation of the prevalence of these disorders and consequently the pattern of risk factors identified, we have used longitudinal data from a Demographic Surveillance System (DSS) in a rural community in northern Malawi [13].

## Methods

### Study population and study area

**Figure 1:**
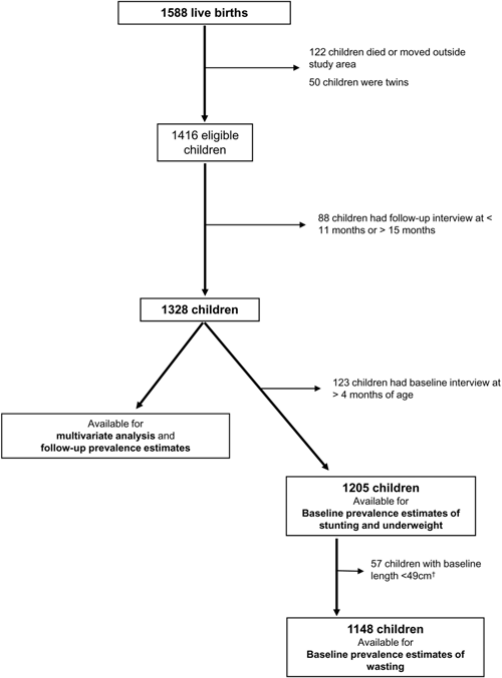
Study design. *Footnote:* ^†^The NCHS reference does not allow for calculation of weight-for-length for children <49 cm

The study was carried out in the southern part of Karonga District, northern Malawi, between August 2002 and October 2004. Out of 1,588 live births recorded in the DSS in the study area, 122 (7.7%) infants either died or left the study area before the follow-up visit, and 50 (3.1%) were excluded for being twins. The analysis of risk factors for malnutrition and prevalence estimates for wasting, underweight and stunting at follow-up included 1328 infants (83.6%) after excluding 88 (5.5%) who had their follow-up visit later than 15 months after birth. The prevalence estimates of underweight and stunting at baseline, when first seen after birth were derived from 1205 infants (75.9%) after excluding 123 (7.7%) infants registered and first assessed more than 120 days after birth. The baseline prevalence estimates of wasting were conducted on 1148 (72.3%) infants after excluding 57 (3.6%) infants with baseline length<49 cm, as the NCHS growth reference is not suitable for calculation of the weight-for-length index for smaller children. Exclusions are summarized in **figure 1**.

### Data collection

Background information on dwelling characteristics, demographic and socio-economic data were drawn from a house-to-house census implemented by trained staff using a standard protocol at the launch of the DSS from August 2002 [13]. Vital events were notified by village informants each responsible for 15–60 households. Notified births were followed by a baseline visit by a project interviewer to formally register the birth and to record the mother's and infant's anthropometric measures and information on feeding practices, health and immunization. A follow-up visit was scheduled 12 months after the birth registration to reassess the child and mother's nutritional status as well as feeding practices, health and immunization. The main caregiver was asked for the approximate age in months when different types of food and beverages were introduced; median age at interview was 12 months (range 11 to 15 months). Throughout the analysis, the term “introduction of water” refers to water and water-based beverages. Complementary food includes breast milk substitutes, cow's milk and maize–based weaning porridges (vernacular: dawale for thin porridge and bara for thick porridge). Family food is defined as all other food items including juices and solid foods.

### Anthropometric measurements and indices

Weight was measured using a spring scale (100 g increments) and length was measured supine using graduated polyurethane plastic mats (0.5 mm increments). Nutritional indices were derived as Z-scores at both time points using the WHO standard and the NCHS reference. Z-scores represent the difference between the height or weight of a child and the median height or weight of the reference population (for the same age and sex) divided by the standard deviation of the reference population.

Global wasting, stunting and underweight were defined as weight-for-length, length-for-age and weight-for-age <-2 z-scores respectively. Maternal nutritional status was assessed using the mid-upper-arm-circumference (MUAC), measured using steel tape (1 mm increments). There is no consensus over the use of MUAC for the classification of adult nutritional status. Cut-offs ranging from 18.5 cm [14] to 22 cm [15] have been proposed to define undernutrition. In our analysis, we used a conservative 21 cm cut-off under which the MUAC has been associated with a Body Mass Index<16 kg/m^2^ in adult women [16], which is widely used by relief agencies for enrolling pregnant and lactating mothers into supplementary feeding programs.

### Data management and statistical analyses

Data were double-entered in MS Access 97. The plausibility of measurements was checked electronically at the point of data entry and implausible values were referred back to the field for confirmation [13].

Calculation of nutritional indices with reference to the WHO standards was done in STATA v.9.2 (StataCorp Ltd, Texas, USA) using a macro provided by WHO [17]. Calculation in reference to the NCHS reference was performed in EpiInfo v.6.04d (Center for Disease Control and Prevention, Georgia, USA). The software manufacturers' default settings were applied regarding cut-offs for biologically improbable values (**Table S1**). Out of range values of z-scores were recoded as missing.

The analyses of risk factors for malnutrition were performed in STATA v.9.2 using logistic regression. The three nutritional indices at 11–15 months were the outcomes. Independent variables with even weak evidence of a crude association (p<0.1) with one of the 3 outcomes in the univariate analysis were eligible for inclusion in the multiple logistic regression analysis, as well as variables that have been identified as risk factors for at least one of the outcomes in other local studies [18], [19], [20]. Housing conditions were measured using a dwelling score based on materials used for building the dwelling. The value of household assets was scored to classify the households into four broad categories of “wealth”. Age at the follow-up interview and sex were kept in the model a priori. Further adjustment for health related variables (vaccination status, hospitalization, history of consulting a traditional healer) was made in final models as these variables could be on the causal pathway between socio-economic variables and malnutrition. Finally, adjustment was made for nutritional status at baseline interview (excluding wasting to avoid co-linearity). The strength of the statistical association was assessed using Wald's test and investigation of potential interactions was performed using Likelihood Ratio Test. The statistical significance of the difference between the prevalence estimates of each outcome calculated with both growth references was assessed using McNemar's z-test.

Multivariate models were built for each of the 3 outcomes calculated with the WHO standards, using a forward stepwise technique. The selected risk factors were then incorporated in models using the outcomes calculated with the NCHS reference. For each outcome, the direction and strength of the associations were then compared between models.

### Ethics

The Karonga DSS called the “Continuous Registration System” was granted ethical approval from the National Health Sciences Research Committee of Malawi and the London School of Hygiene and Tropical Medicine Ethics Committee. Heads of participating households gave verbal consent for being included in the DSS.

A further application to use the DSS data for the present study was granted approval from the London School of Hygiene and Tropical Medicine Ethics Committee. The committee accepted the initial verbal consent since it would have been impossible to get a written consent from the guardians of each individual infant included in this analysis due to vital events and population movements.

## Results

### Sample characteristics

The main characteristics of the sample are described in **Table 1**. Households were headed by males in 88% of cases, with an average age of nearly 40 years of whom three quarters were self-employed, mainly in farming and fishing. This activity however was the primary source of income for only half of the households. Access to improved source of drinking water was widespread (80%) but not to electricity (2.4%). Nearly every household cultivated a median [Interquartile Range] 2.0 [1.0–3.0] acres of land. Cassava was the most frequently cultivated crop (96.5%) followed by maize (88.4%), rice (41.3%), potatoes (31.2%) and groundnuts (28.7%).

**Table 1:** Main characteristics of the sample population.

Demography and education	n/N	%*
Male	685/1,328	51.6%
Maternal age at birth of infant in years (mean±SD†)	1,328	25.6±6.3
Infant age at baseline in days (median [IQR]‡)	1,328	28 [16–53]
Infant age at follow-up in days (mean±SD†)	1,328	384±32
Maternal education level≥primary (8 years)	666/1,294	51.5%
Father's education level≥primary (8 years)	948/1,235	76.8%
**Health**		
Vaccination coverage at follow-up		
BCG	1,255/1,328	94.5%
Measles	1,093/1,328	82.3%
Polio 3	1,220/1,328	91.9%
DPT 3	1,231/1,328	92.7%
**Socio-economic status**		
Dwelling score (housing conditions)$		
1 (best)	198/1,309	15.1%
2	173/1,309	13.2%
3	416/1,309	31.8%
4 (worst)	522/1,309	39.9%
Asset score: Possessions value in US Dollars§		
<5	277/1,328	20.9%
5–9.99	262/1,328	19.7%
10–49.99	460/1,328	34.6%
≥50	329/1,328	24.8%
HH main source of income		
Employment & letting	210/1,309	16.0%
Piecework & gathering	141/1,309	10.8%
Farming	514/1,309	39.3%
Fishing	134/1,309	10.2%
Trade (small scale)	175/1,309	13.4%
Selling own manufactured goods or food/beverage	61/1,309	4.7%
Other	74/1,309	5.6%
**Nutrition**		
Duration of exclusive breastfeeding (mean±SD†) in months	1,328	4±2
Age introduction of water (mean±SD†) in months	1,324	5±2
Age intro complementary food (mean±SD†) in months	1,321	5±2
Age intro of family food (mean±SD†) in months	1,296	8±2

* Percentage or otherwise specified

† Mean±standard deviation

‡ Median [Interquartile Range]

$ Score based on materials used for the roof, walls and floor

§ Score based on possession of 8 items (Yes = 1; No = 0 for motorbike, oxcart, bicycle, clock, radio, canoe, fishnet and mosquito net), plus cattle categorized by number of head (0 = 0; 1 = 1; 2/3 = 2; 4/6 = 3; 7+ = 4). The score is then converted in monetary value of possessions.

Households in the DSS area were located on average within 1 km radius of a static health facility or mobile location where under-5-clinics were provided. By the time of the follow-up visit, 19.1% of the children in the study population had had at least one hospital/health center admission, and 28.6% had been to a traditional healer at least once. The vaccination coverage for tuberculosis (94.5%), poliomyelitis 3 (91.9%), Diphtheria-Pertusis-Tetanus 3 (92.7%) and measles (82.3%) was above the national average, which stands at 78%, 79%, 64% and 69% respectively for the year 2004 [21]. Virtually all the mothers (99%) had attended antenatal clinics at least once during pregnancy.

Maternal malnutrition at both baseline and follow-up interviews was very low at 2.1% and 1.4% respectively. By the age of 4 months, 17.8% of children were given water in addition to breast milk and 23.5% were given complementary foods. The median duration of exclusive breastfeeding was 4 months and 40.5% of infants were still exclusively breast fed until the end of the 5^th^ month of life as recommended by WHO.

### Prevalence of malnutrition

**Figure 2:**
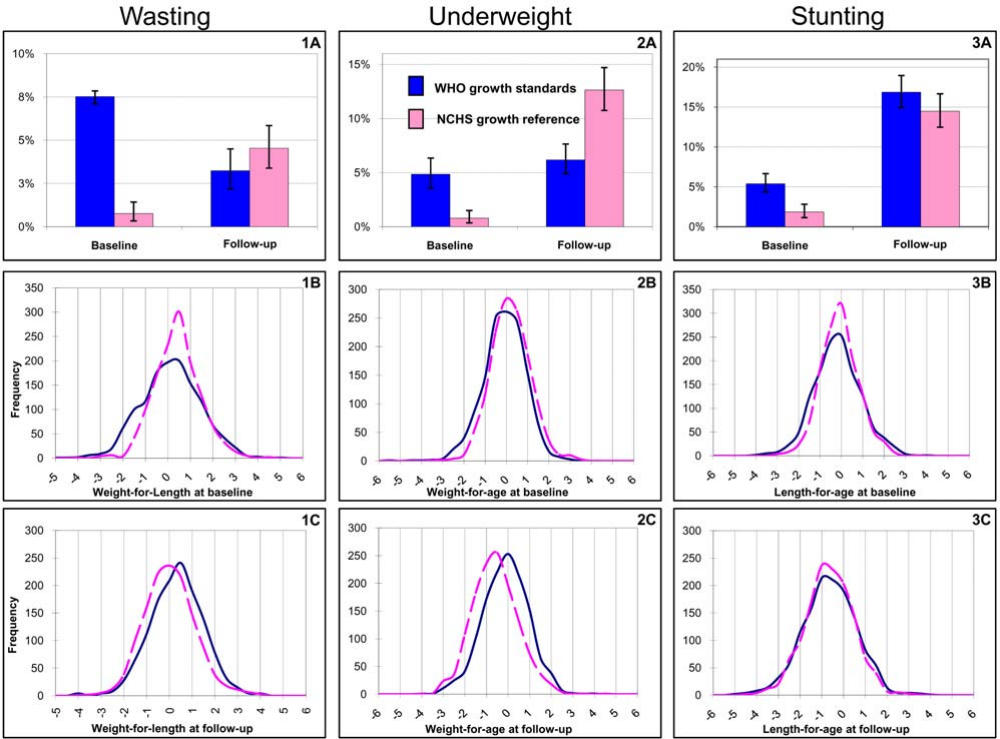
A - Prevalence *[95% confidence intervals]* of wasting (1A), underweight (1B) and stunting (1C) according to the growth reference used. B–C - Frequency distribution of nutrition indices in z-score at baseline (B) and follow-up (C) according to the growth reference used. The prevalence of malnutrition reported in (A) corresponds to the surface under the curve below -2 z-score.

As illustrated in **figure 2** (2.1a to 2.3a), there were considerable differences in the estimated prevalence of malnutrition at baseline (0–4 months) according to which growth reference was used. The prevalence estimates were 2.9, 6.1, and 8.5-fold higher for stunting, underweight and wasting respectively using the WHO standards (p<0.001 for all). At follow-up, there was very weak evidence of an increased proportion of stunted infants (p = 0.09; figure 2.3a) and lower proportion of wasted infants (p = 0.10; figure 2.1a) with the WHO-based estimates compared to that of the NCHS estimates. However the estimated prevalence of underweight (figure 2.2a) was half that assessed using the NCHS reference (p<0.001).

At baseline, the WHO standards-based distribution curve of weight-for-length was flatter than the NCHS-based distribution and slightly shifted toward negative values of z-score (difference between means is 0.26 z-scores), leading to 8.5 times more infants being classified as wasted when using the WHO standards (7.5% vs. 0.9%; figure 2.1a; 2.1b). This pattern was not observed after 11 months (figure 2.1c); the curves were similar in shape with a slight shift toward positive values of z-scores for the WHO standards-based distribution curve and little change in the proportion of children with weight-for-length<-2 z-score (3.3% and 4.5% for the WHO standards and NCHS reference-based prevalence respectively ; figure 2.1a to 2.1c).

For underweight the shapes of the distribution curves with the two growth references were similar to each other at both time-points. However, compared to the NCHS reference-based distribution curve, at baseline the curve based on the WHO standards was shifted to the left (difference in means 0.26 z-score) and that at follow-up to the right (difference in means 0.54 z-score; figure 2.2b to 2.2c). The WHO standards gave a prevalence of underweight 3.6 times higher early in infancy (6.1% against 1.7%) and half the estimated prevalence of the NCHS reference in the second half of infancy (6.6% against 13.6% ; figure 2.2a).

A flatter curve with the WHO standards led to a 2-fold increase in the prevalence of stunting at baseline (8.1% and 4.1% for the WHO and NCHS reference based prevalence respectively), but the curves based on the two standards were similar at follow-up time (figure 2-3a to 2–3c). The sample's mean length-for-age was nearly equal with both growth references and at both time-points.

### Risk factors for malnutrition

Crude associations between various risk factors and wasting, underweight and stunting at 11–15 months, calculated using the WHO standards, are shown in **table S2**.

Other variables (sex of the head of household, maternal education and nutritional status, distance from health center or vaccine status at 11–15 months, land area cultivated by the household, and growing cassava, rice, potatoes or groundnut) were not associated with any of the outcomes (not shown).

Those factors that remained significant in multivariate models for at least one of the outcomes are shown in **table 2**. No evidence of interaction was found between any of the independent factors. Following evidence of a linear trend across categories for 2 of the three outcomes (p = 0.02 for wasting and underweight) the dwelling score was included as a linear variable in the models.

**Table 2:** Multivariate analysis of early risk factors for adverse anthropometric status at 11–15 months based on either WHO or NCHS growth reference†

	WASTING		UNDERWEIGHT		STUNTING	
	WHO (n = 1230)	NCHS (n = 1230)	WHO (n = 1237)	NCHS (n = 1237)	WHO (n = 1230)	NCHS (n = 1231)
DEMOGRAPHIC	*OR [95% CI]*	*OR [95% CI]*	*OR [95% CI]*	*OR [95% CI]*	*OR [95% CI]*	*OR [95% CI]*
***Age of head of household (years)***						
<25	2.67 *[1.06–6.74] * **	2.08 *[0.94–4.06] * *	0.88 *[0.38–2.07]*	1.08 *[0.62–1.90]*	0.92 *[0.55–1.55]*	1.00 *[0.59–1.71]*
25–39	ref	ref	ref	ref	ref	ref
40–54	1.61 *[0.59–4.43]*	0.80 *[0.31–2.07]*	0.81 *[0.39–1.69]*	0.69 [0.41–1.17]	0.76 *[0.48–1.19]*	0.78 *[0.48–1.28]*
55 +	1.38 *[0.49–3.91]*	1.18 *[0.49–2.82]*	1.31 *[0.66–2.59]*	1.04 *[0.62–1.74]*	1.31 *[0.85–2.02]*	1.37 *[0.87–2.15]*
***Maternal age (years)***						
<20	1.02 *[0.46–2.28]*	0.83 *[0.40–1.71]*	0.84 *[0.42–1.67]*	1.53 *[0.97–2.40] * *	1.22 *[0.82–1.83]*	1.36 *[0.89–2.07]*
20–29	ref	ref	ref	ref	ref	ref
30–39	0.26 *[0.07–0.97] * **	0.59 *[0.24–1.46]*	1.15 *[0.61–2.19]*	1.20 *[0.75–1.92]*	1.06 *[0.71–1.59]*	1.10 *[0.71–1.70]*
40 +	1.70 *[0.29–10.02]*	0.99 *[0.11–8.96]*	4.80 *[1.43–16.12] * **	2.80 *[0.93–8.49] * *	1.35 *[0.46–3.97]*	1.70 *[0.57–5.11]*
***Season of birth***						
Warm & rainy (Jan–May)	ref	ref	ref	ref	ref	ref
Cool & dry (June–Sept)	1.46 *[0.69–3.10]*	1.04 *[0.55–1.98]*	1.33 *[0.78–2.30]*	0.80 *[0.54–1.19]*	1.03 [0.73–1.45]	1.18 *[0.82–1.70]*
Dry (Oct–Dec)	0.95 *[0.35–2.55]*	0.87 *[0.39–1.96]*	1.10 *[0.55–2.22]*	1.23 *[0.77–1.95]*	1.56 *[1.03–2.35] * **	1.61 *[1.04–2.50] * **
**SOCIOECONOMIC**						
***Source of drinking water***						
Tap	ref	ref	ref	ref	ref	ref
Bore hole	1.77 *[0.57–6.62]*	1.31 *[0.46–3.75]*	3.14 *[1.04–9.44] * **	1.83 *[0.94–3.58] * *	1.62 *[0.96–2.75] * *	1.45 *[0.82–2.56]*
River or lake	5.61 *[1.37–22.97] * **	3.43 *[1.11–10.61] * **	4.59 *[1.41–14.99] * **	2.38 *[1.14–4.96] * **	1.78 *[0.98–3.22] * *	1.61 *[0.85–3.06]*
***Asset score (USD)***						
> = 50	ref	ref	ref	ref	ref	ref
10–49.99	4.44 *[1.21–16.38] * **	3.25 *[1.25–8.42] * **	1.34 *[0.66–2.73]*	0.91 *[0.57–1.44]*	1.01 *[0.67–1.52]*	1.01 *[0.65–1.56]*
5–9.99	4.37 *[1.12–16.99] * **	3.22 *[1.16–8.99] * **	1.61 *[0.75–3.48]*	0.86 *[0.50–1.46]*	1.17 *[0.73–1.85]*	1.22 *[0.74–2.00]*
<5	2.65 *[0.67–10.54]*	2.04 *[0.71–5.89]*	1.30 *[0.60–2.82]*	0.80 *[0.47–1.35]*	1.19 *[0.76–1.87]*	1.25 *[0.78–2.01]*
***Households***' ***main source of income***						
Farming	ref	ref	ref	ref	ref	ref
Employment & letting	2.62 *[0.84–8.15] * *	2.59 *[0.97–6.95] * *	1.58 *[0.72–3.47]*	0.83 *[0.45–1.54]*	0.74 *[0.44–1.24]*	0.50 *[0.27–0.92] * **
Piecework & gathering	4.07 *[1.60–10.33] * **	3.10 *[1.30–7.35] * **	2.46 *[1.25–4.85] * **	2.01 *[1.20–3.36] * **	1.16 *[0.71–1.89]*	1.10 *[0.66–1.84]*
Fishing	0.39 *[0.05–3.24]*	2.06 *[0.72–5.87]*	0.36 *[0.10–1.29]*	0.42 *[0.20–0.88] * **	0.52 *[0.29–0.94] * **	0.56 *[0.31–1.04] * *
Trade	1.53 [*0.51–4.64]*	1.65 *[0.64–4.25]*	1.07 *[0.48–2.37]*	1.30 *[0.77–2.22]*	0.93 *[0.58–1.50]*	0.96 *[0.58–1.57]*
Selling own goods or snacks	2.73 [*0.68–11.04]*	1.19 *[0.25–5.65]*	1.18 *[0.38–3.69]*	1.24 *[0.66–2.75]*	0.95 *[0.46–1.96]*	1.08 *[0.52–2.24]*
Other	2.90 *[0.81–10.35]*	3.66 *[1.27–10.53] * **	1.16 *[0.39–3.41]*	1.25 *[0.58–2.68]*	1.04 *[0.54–2.00]*	1.09 *[0.56–2.13]*
***Father***'***s education level***						
Secondary or tertiary	ref	ref	ref	ref	ref	ref
Completed primary	0.87 *[0.37–2.04]*	0.81 *[0.39–1.68]*	0.95 *[0.52–1.73]*	1.20 *[0.77–1.85]*	1.45 *[1.00–2.12] * *	1.27 *[0.85–1.90]*
None or uncompleted primary	1.16 *[0.48–2.79]*	1.29 *[0.62–2.68]*	1.17 *[0.61–2.24]*	1.87 *[1.18–2.94] * **	1.73 *[1.14–2.60] * **	1.81 *[1.17–2.77] * **
Unknown	0.83 *[0.17–4.03]*	0.60 *[0.13–2.76]*	0.56 *[0.15–2.02]*	0.49 *[0.25–1.31]*	0.96 *[0.49–1.89]*	0.82 *[0.39–1.73]*
**AGRICULTURE**						
***Growing maize***						
Yes	1.06 *[0.41–2.73]*	1.44 *[0.62–3.38]*	0.56 *[0.30–1.01] * *	0.71 *[0.45–1.12]*	0.67 *[0.45–1.00] * **	0.73 *[0.47–1.11]*
No	ref	ref	ref	ref	ref	ref
NUTRITION						
***Maternal malnutrition at follow-up***						
Yes	2.17 *[0.24–19.79]*	1.01 *[0.12–8.38]*	10.79 *[3.26–35.74]* ***	3.68 [*1.21–11.16] * **	2.38 *[0.79–7.20]*	1.87 *[0.58–6.01]*
No	ref	ref	ref	ref	ref	ref

* p<0.1 ;

** p<0.05 ;

*** p<0.001

Note: 1328 records were included in the models. Infants with one or more missing value for variables in the model were not accounted for.

† Adjustment made for all other variables in the table, plus age of introduction of water, complementary and family food, dwelling score, sex and age at follow-up interview

Infants from households with young heads, getting their drinking water from river or lake, with low household assets, and primary income source as piecework/gathering or employment/letting were more likely to be wasted. No other feeding practice showed evidence of association with wasting after adjusting for all other variables in the model. Those with mothers aged 30–39 were less likely to be wasted than those with mothers aged 20–29.

Being underweight at follow-up was significantly associated with high maternal age and maternal malnutrition at follow-up, not getting water from a tap, and household income from piecework/gathering. Growing maize was weakly associated with a 44% protective effect.

Being stunted at follow-up was associated with birth in the dry season and low paternal education level. There was also weak evidence of an association with not getting water from a tap (p<0.1). Males were 1.6 times more likely to be stunted than females (p = 0.003) and older infants at follow-up were also more likely to be stunted then those who were younger when seen (p = 0.002; not shown). A household income from fishing was significantly protective, as was growing maize.

Stunting at baseline was strongly associated with underweight and stunting at follow-up (p = 0.001 and p<0.001 respectively), and being underweight at baseline was strongly associated with all 3 outcomes at follow-up (p<0.001 except wasting where p = 0.005). Adjusting for vaccination status, hospital admission or traditional healer visits, and for stunting and underweight at baseline made little difference to the associations with other variables shown in table 2.

### Comparing WHO standards-based and NCHS reference-based models

Overall there was a good agreement in the pattern of risk factors between the models based on the two references and poor growth at follow-up, although differences existed in terms of the strength of associations. The associations between explanatory variables and wasting and stunting were on average stronger in the WHO standards-based models than the NCHS reference-based models but the directions of the associations were preserved. This is exemplified by the increased risk of wasting in children living in households with young heads identified in the WHO standards-based model. Evidence of this association in the NCHS reference-based model was weaker (p = 0.07) but the direction and scale of the effect was consistent (Odds Ratio (OR)_NCHS_ = 2.08 vs. OR_WHO_ = 2.67).

A higher estimated prevalence of underweight at follow-up based on the NCHS reference meant an increased power in the NCHS reference-based multivariate analysis than in the WHO standards-based model. For this outcome, more risk factors were significantly associated with underweight at follow-up in the NCHS reference-based model including having a young mother (<20 years old), low father's education level, and being older at follow-up interview (OR_NCHS_ = 1.01, p = 0.03; not shown). There was good evidence that fishing as a source of income was protective (p = 0.02) although no evidence of this association could be detected in the WHO-based model (p = 0.12). Conversely there was weak evidence that growing maize was protective for underweight in the WHO-based model (p = 0.06) but not in the NCHS-based one (p = 0.14).

## Discussion

Using the new WHO growth standards increased the estimated prevalence of malnutrition in early infancy by a factor 3 to 8.5 depending on the index under consideration. This difference was not found at one year of age but the underweight estimate was halved compared to that obtained with the NCHS reference.

The direction of the differences in prevalence at both time-points are consistent with previous findings but their magnitude was larger in our study [7], [8]. One can expected an increase in the prevalence of malnutrition from switching to the WHO standards, but the magnitude of this increase depends on the nutritional status of the population under consideration and should not be assumed from previous studies. This is illustrated by figure 2.1b-2.3b where the WHO standards-based distribution curves for the three outcomes are not only shifted towards negative values of z-scores, but also flatter compared to the NCHS reference-based distribution curves.

Malnutrition in early infancy is seldom addressed in therapeutic feeding programs as it conflicts with current recommendations on exclusive breastfeeding [22] and requires highly skilled medical personnel. If WHO standards are to be adopted, the dramatic increase in the number of infants diagnosed as wasted may warrant a scaling-up of antenatal supplementation programs including multiple micronutrients to address small birth size, one of the strongest determinants of growth failure at 12 months in Malawi [18]. However interventions directly targeting infants should not be ruled out providing there is adequate medical surveillance. In addition, there is certainly a need to develop further therapeutic strategies and products adapted to the specific needs of this age group.

The surge in the proportion of infants diagnosed as malnourished attributable to the use of the WHO standards should not hamper the need to find which reference better characterizes malnutrition needing intervention. It is important to know if the sensitivity of the cut-offs that were applied to define malnutrition with the NCHS reference (-2 z-scores) are still appropriate with the WHO standards for predicting poor outcomes.

Although the measured prevalence of malnutrition was different using the two references, there was generally good agreement on the pattern of risk factors for growth faltering in infancy. The stronger associations between risk factors and wasting and stunting in the WHO standards-based model and conversely with underweight in the NCHS reference-based model were partly due to increased power.

In our study it was not possible to obtain weight and length at birth, for logistic reasons. Instead we used anthropometric status within 0–4 months as a proxy indicator. Hence low birth weight and small for gestational age babies which have different growth trajectories in infancy may have affected the results. Earlier studies in Malawi and elsewhere suggest that, in normal birth weight babies, although height faltering may be present at birth, weight faltering does not start before age 3 to 4 months [23], [24]. The study population was slightly above national average estimates for health/nutrition and socio-economic status [21]. Recent studies in southern Malawi among children aged 12–18 months have estimated prevalences of malnutrition ranging from 40%–46% underweight, 2%–8% wasting and 46%–71% stunting when using the NCHS reference [18], [25]. Malawi has been hard hit by the HIV epidemic and Karonga has had a stable HIV prevalence of around 10% in the adult population [26]. While direct estimates for children are not available, <3% of children are likely to be infected vertically [27]. Orphanhood was rare in this age group (3 maternal, 11 paternal, non double, by the time of the follow-up interview), therefore unlikely to affect our results.

Since the new WHO growth standards are based on optimal growth patterns and breast-fed infants they are to be welcomed. The similarity of the risk factors identified with each growth reference is an additional argument in favour of adopting the new WHO growth standards. However our results suggest that considerable caution will be needed in comparing prevalence estimates measured with WHO standards and those from older studies relying on the NCHS reference. Ideally, results should be presented with both references. Whether the young infants additionally identified as malnourished by this new standard are more appropriate targets for interventions than those identified with the NCHS reference, and whether such interventions can change their growth trajectories, requires further study.

## Supporting Information

Table S1 Cut-offs used to exclude biologically implausible values for weight-for-height, weight-for-age, and length-for-age in z-scores as defined by the software manufacturer. Outliers were recoded as missing in the analysis.
(0.08 MB DOC)

Table S2 Crude associations between demographic and socio-economic factors, health, feeding practices, and anthropometric status at baseline and wasting, stunting and underweight at follow-up calculated with the WHO Growth Standards.
(0.16 MB DOC)
