# Supplementary material for: Implication of New WHO Growth Standards on Identification of Risk Factors and Estimated Prevalence of Malnutrition in Rural Malawian Infants
Source: PLoS One. 2008 Jul 16;3(7):e2684. doi: 10.1371/journal.pone.0002684 (PMC2442189; doi:10.1371/journal.pone.0002684)
Supplement: Table S1 — Cut-offs used to exclude biologically implausible values for weight-for-height, weight-for-age, and length-for-age in z-scores as defined by the software manufacturer. Outliers were recoded as missing in the analysis. (0.08 MB DOC) [file pone.0002684.s001.doc]

Table S1: Cut-offs used to exclude biologically implausible values for weight-for-height, weight-for-age, and length-for-age in z-scores as defined by the software manufacturer. Outliers were recoded as missing in the analysis.

|  | **NCHS Reference^†^** | | **WHO Growth standards^‡^** | |
| --- | --- | --- | --- | --- |
| ***Z-scores*** | ***min*** | ***max*** | ***min*** | ***max*** |
| Weight-for-Length | < - 4 | > + 6 | < - 5 | > + 5 |
| Length-for-Age | < - 6 | > + 6 | < - 6 | > + 6 |
| Weight-for-Age | < - 6 | > + 6 | < - 6 | > + 5 |

^†^ Using EpiInfo 6.04d, Center for Disease Prevention and Control (CDC), Atlanta, GA, USA

^‡^ Using STATA 9, World Health Organization, Geneva, Switzerland

Table S2: Crude associations between selected risk factors and wasting, stunting and underweight at follow-up calculated with the WHO Growth Standards

|  | **Wasting at 11-15 months** | | **Underweight at 11-15 months** | | **Stunting at 11-15 months** | |
| --- | --- | --- | --- | --- | --- | --- |
| **DEMOGRAPHY** | ***n/N (%)*** | ***OR [95% CI]*** | ***n/N (%)*** | ***OR [95% CI]*** | ***n/N (%)*** | ***OR [95% CI]*** |
| Sex  Female  Male | 17/637 (2.7)  26/682 (3.8) | ref  1.44 [0.78 - 2.69] | 33/642 (5.1)  54/684 (7.9) | ref  1.58 [1.01 - 2.47] ** | 92/637 (14.4)  152/683 (22.2) | ref  1.70 [1.28 - 2.26] *** |
| Maternal age at birth (years)  < 20  20 - 29  30 - 39  40 + | 13/270 (4.8)  24/737 (3.3)  4/282 (1.4)  2/30 (6.7) | 1.50 [0.75 - 3.00]  ref  0.43 [0.15 - 1.24]  2.12 [0.48 - 9.43] | 15/273 (5.5)  45/740 (6.1)  21/283 (7.4)  6/30 (20.0) | 0.90 [0.49 - 1.64]  ref  1.24 [0.72 - 2.12]  3.86 [1.50 - 9.92] ** | 52/270 (19.2)  133/738 (18.0)  52/282 (18.4)  7/30 (23.3) | 1.09 [0.76 - 1.54]  ref  1.03 [0.76 - 1.55]  1.38 [0.58 - 3.29] |
| Age of head of household at birth (years)  < 25  25 - 39  40 - 54  55 + | 10/149 (6.7)  18/685 (2.6)  8/255 (3.1)  7/230 (3.0) | 2.67 [1.20 - 5.90] **  ref  1.20 [0.51 - 2.79]  1.16 [0.48 - 2.82] | 8/150 (5.3)  47/688 (6.8)  15/257 (5.8)  17/231 (7.4) | 0.77 [0.36 - 1.66]  ref  0.84 [0.46 - 1.54]  1.08 [0.61 - 1.93] | 27/149 (18.1)  128/687 (18.6)  39/255 (15.3)  50/229 (21.8) | 0.97 [0.61 - 1.53]  ref  0.79 [0.53 - 1.17]  1.22 [0.84 - 1.76] |
| Infant season of birth  Warm & rainy (Jan – May)  Cool & dry (Jun – Sept)  Dry (Oct – Dec) | 16/532 (3.0)  20/545 (3.7)  7/242 (2.9) | ref  1.23 [0.63 - 2.40]  0.96 [0.39 - 2.37] | 31/535 (5.8)  39/549 (7.1)  17/242 (7.0) | ref  1.24 [0.76 - 2.02]  1.23 [0.67 - 2.27] | 93/531 (17.5)  95/547 (17.4)  56/242 (23.1) | ref  0.99 [0.72 - 1.36]  1.42 [0.98 - 2.06] * |
| **SOCIO-ECONOMIC STATUS** |  |  |  |  |  |  |
| Father's education level  Unknown  None or uncompleted primary  Completed primary  Secondary or tertiary | 2/93 (2.1)  12/285 (4.2)  14/434 (3.2)  15/507 (3.0) | 0.72 [0.16 - 3.21]  1.44 [0.67 - 3.12]  1.09 [0.52 - 2.29]  ref | 3/93 (3.2)  25/287 (8.7)  30/436 (6.9)  29/510 (5.7) | 0.55 [0.16 - 1.85]  1.58 [0.91 - 2.76]  1.23 [0.72 - 2.08]  ref | 14/93 (15.1)  68/284 (23.9)  85/434 (19.6)  77/509 (15.1) | 0.99 [0.54 - 1.84]  1.77 [1.23 - 2.54] **  1.37 [0.97 - 1.92] *  ref |
| Source of drinking water  Tap  Bore hole  River or lake | 3/200 (1.5)  25/846 (2.9)  15/268 (5.6) | ref  2.00 [0.60 - 6.69]  3.89 [1.11 - 13.64] ** | 5/200 (2.5)  59/853 (6.9)  23/268 (8.6) | ref  2.90 [1.15 - 7.32] **  3.66 [1.37 - 9.81] ** | 26/201 (12.9)  160/847 (18.9)  57/267 (21.3) | ref  1.57 [1.00 - 2.45] **  1.83 [1.10 - 3.03] ** |
| Dwelling category  1 (best)  2  3  4 (worst)  Trend across categories | 2/197 (1.0)  5/172 (2.9)  12/412 (2.9)  24/519 (4.6) | ref  2.92 [0.56 - 15.24]  2.93 [0.65 - 13.20]  4.72 [1.11 - 20.19] **  1.51 [1.07 – 2.14] ** | 6/197 (3.0)  10/173 (5.8)  28/416 (6.7)  43/521 (8.2) | ref  1.95 [0.69 - 5.49]  2.30 [0.94 - 5.64] *  2.86 [1.20 - 6.84] **  1.34 [1.06 – 1.68] ** | 34/198 (17.3)  26/172 (15.1)  76/412 (18.4)  104/519 (20.0) | ref  0.86 [0.49 - 1.50]  1.09 [0.70 - 1.70]  1.21 [0.79 - 1.85]  1.09 [0.95 – 1.25] |
| Asset score (USD)  < 5  5 - 9.99  10 - 49.99  >= 50 | 10/275 (3.6)  12/261 (4.6)  17/456 (3.7)  4/327 (1.2) | 3.05 [0.94 - 9.83] *  3.89 [1.24 - 12.21] **  3.13 [1.04 - 9.38] **  ref | 20/277 (7.2)  20/262 (7.6)  32/458 (7.0)  15/329 (4.6) | 1.63 [0.82 - 3.25]  1.73 [0.87 - 3.45]  1.57 [0.84 - 2.95]  ref | 60/275 (21.8)  49/261 (18.8)  79/457 (17.3)  56/327 (17.1) | 1.35 [0.90 - 2.03]  1.12 [0.73 - 1.71]  1.01 [0.69 – 1.47]  ref |
| HH main source of income  farming  employment & letting  piecework & gathering  fishing  trade  Selling own goods and snacks  other | 13/513 (2.5)  6/209 (2.9)  10/138 (7.2)  1/133 (0.7)  5/173 (2.9)  3/60 (5.0)  4/74 (5.4) | ref  1.13 [0.43 - 3.03]  3.00 [1.29 - 7.01] **  0.29 [0.04 - 2.25]  1.14 [0.40 - 3.26]  2.02 [0.56 - 7.32]  2.20 [0.70 - 6.93] | 34/514 (6.6)  12/210 (5.7)  17/141 (12.0)  3/134 (2.2)  11/173 (6.3)  4/61 (6.6)  5/74 (6.8) | ref  0.86 [0.43 - 1.69]  1.93 [1.05 - 3.58] **  0.32 [0.10 - 1.07] *  0.96 [0.47 - 1.93]  0.99 [0.34 - 2.89]  1.02 [0.39 - 2.70] | 105/513 (20.5)  28/209 (13.4)  33/138 (23.9)  17/133 (12.8)  33/175 (18.9)  11/59 (18.6)  15/74 (20.3) | ref  0.60 [0.38 - 0.94] **  1.22 [0.78 - 1.91]  0.57 [0.33 - 0.99] **  0.90 [0.58 - 1.40]  0.89 [0.45 - 1.77]  0.99 [0.54 - 1.81] |
| **AGRICULTURE** |  |  |  |  |  |  |
| Growing maize  No  Yes | 7/205 (3.4)  36/1109 (3.2) | ref  0.95 [0.42 - 2.16] | 19/205 (9.3)  68/1116 (6.1) | ref  0.64 [0.37 – 1.08] * | 44/205 (21.5)  199/1110 (17.9) | ref  0.80 [0.55 - 1.15] |
| **HEALTH** |  |  |  |  |  |  |
| History of hospital admission  No  Yes | 3/1061 (0.3)  12/249 (4.8) | ref  1.68 [0.85 - 3.32] | 60/1066 (5.6)  26/251 (10.4) | ref  1.94 [1.20 - 3.14] ** | 194/1061 (18.3)  48/250 (19.2) | ref  1.06 [0.74 - 1.51] |
| History of traditional healer consultation  No  Yes | 28/928 (3.0)  15/373 (4.0) | ref  1.35 [0.71 - 2.55] | 45/934 (4.8)  42/374 (11.2) | ref  2.50 [1.61 - 3.88] *** | 166/929 (17.9)  77/373 (20.6) | ref  1.20 [0.88 - 1.61] |
| **NUTRITION & ANTHROPOMETRY** |  |  |  |  |  |  |
| Age introduction of water  < 4 months  4-5 months  ≥ 6 months | 13/235 (5.5)  11/388 (2.8)  19/692 (2.7) | 2.07 [1.01 – 4.27] **  1.03 [0.49 – 2.19]  ref | 22/236 (9.3)  24/389 (6.2)  41/697 (5.9) | 1.64 [0.96 - 2.82] *  1.05 [0.63 - 1.77]  ref | 46/234 (19.7)  74/389 (19.0)  124/693 (17.9) | 1.12 [0.77 - 1.64]  1.08 [0.78 - 1.48]  ref |
| Age introduction of complementary food  < 4 months  4-5 months  ≥ 6 months | 16/310 (5.2)  8/332 (2.4)  18/671 (2.7) | 1.97 [0.99 - 3.93] *  0.90 [0.39 - 2.08]  ref | 28/310 (9.0)  22/333 (6.6)  36/677 (5.3) | 1.77 [1.06 - 2.95] **  1.26 [0.73 - 2.18]  ref | 62/309 (20.1)  64/332 (19.3)  117/672 (17.4) | 1.19 [0.85 - 1.68]  1.13 [0.81 - 1.59]  ref |
| Age introduction of family food  < 6 months  ≥ 6 months | 4/48 (8.3)  39/1240 (3.1) | 2.80 [0.96 - 8.17] *  ref | 5/48 (10.4)  78/1247 (6.2) | 1.74 [0.67 - 4.52]  ref | 12/48 (25.0)  226/1240 (18.2) | 1.50 [0.77 - 2.92]  ref |
| Wasting at baseline  no  moderate  severe | 38/1182 (3.2)  3/77 (3.9)  1/21 (4.8) | ref  1.23 [0.37 - 4.06]  1.51 [0.20 - 11.56] | 67/1189 (5.6)  15/77 (19.5)  4/21 (19.0) | ref  4.07 [2.20 - 7.53] ***  3.96 [1.30 - 12.09] ** | 216/1189 (18.2)  15/76 (19.7)  4/21 (19.0) | ref  1.11 [0.62 - 1.99]  1.06 [0.35 - 3.18] |
| Stunting at baseline  no  moderate  severe | 33/1182 (2.8)  7/88 (7.9)  3/25 (12.0) | ref  3.01 [1.29 - 7.01] **  4.75 [1.35 - 16.65] ** | 61/1189 (5.1)  16/88 (18.2)  10/25 (40.0) | ref  4.11 [2.26 - 7.49] ***  12.33 [5.32 - 28.57] *** | 33/1182 (2.8)  32/87 (36.8)  17/25 (68.0) | ref  3.06 [1.92 - 4.87] ***  11.19 [4.76 - 26.29] *** |
| Underweight at baseline  no  moderate  severe | 31/1227 (2.5)  8/68 (11.8)  4/16 (25.0) | ref  5.14 [2.27 - 11.67] ***  12.86 [3.93 - 42.12] *** | 55/1234 (4.5)  24/68 (35.3)  8/16 (50.0) | ref  11.69 [6.64 - 20.59] ***  21.44 [7.76 - 59.24] *** | 199/1229 (16.2)  34/68 (50.0)  9/15 (60.0) | ref  5.18 [3.14 - 8.52] ***  7.76 [2.73 - 22.05] *** |
| Maternal malnutrition at follow-up  Yes  No | 1/43 (2.3)  17/1263 (1.3) | 1.75 [0.23 - 13.42]  ref | 6/86 (7.0)  12/1227 (1.0) | 7.59 [2.78 - 20.76] ***  ref | 6/241 (2.5)  12/1066 (1.1) | 2.24 [0.83 - 6.03]  ref |

* p< 0.1 ; ** p< 0.05 ; *** p< 0.001
