# Supplementary material for: Implication of New WHO Growth Standards on Identification of Risk Factors and Estimated Prevalence of Malnutrition in Rural Malawian Infants
Source: PLoS One. 2008 Jul 16;3(7):e2684. doi: 10.1371/journal.pone.0002684 (PMC2442189; doi:10.1371/journal.pone.0002684)
Supplement: Table S2 — Crude associations between demographic and socio-economic factors, health, feeding practices, and anthropometric status at baseline and wasting, stunting and underweight at follow-up calculated with the WHO Growth Standards. (0.16 MB DOC) [file pone.0002684.s002.doc]

Table S2: Crude associations between demographic and socio-economic factors, health, feeding practices, and anthropometric status at baseline and wasting, stunting and underweight at follow-up calculated with the WHO Growth Standards

|  | **WASTING** | | **UNDERWEIGHT** | | **STUNTING** | |
| --- | --- | --- | --- | --- | --- | --- |
| **DEMOGRAPHY** | ***n/N (%)*** | ***OR [95% CI]*** | ***n/N (%)*** | ***OR [95% CI]*** | ***n/N (%)*** | ***OR [95% CI]*** |
| ***Sex*** |  |  |  |  |  |  |
| Female | 17/637 (2.7) | ref | 33/642 (5.1) | ref | 92/637 (14.4) | ref |
| Male | 26/682 (3.8) | 1.44 [0.78 - 2.69] | 54/684 (7.9) | 1.58 [1.01 - 2.47] ** | 152/683 (22.2) | 1.70 [1.28 - 2.26] *** |
| ***Maternal age at birth (years)*** |  |  |  |  |  |  |
| < 20 | 13/270 (4.8) | 1.50 [0.75 - 3.00] | 15/273 (5.5) | 0.90 [0.49 - 1.64] | 52/270 (19.2) | 1.09 [0.76 - 1.54] |
| 20 - 29 | 24/737 (3.3) | ref | 45/740 (6.1) | ref | 133/738 (18.0) | ref |
| 30 - 39 | 4/282 (1.4) | 0.43 [0.15 - 1.24] | 21/283 (7.4) | 1.24 [0.72 - 2.12] | 52/282 (18.4) | 1.03 [0.76 - 1.55] |
| 40 + | 2/30 (6.7) | 2.12 [0.48 - 9.43] | 6/30 (20.0) | 3.86 [1.50 - 9.92] ** | 7/30 (23.3) | 1.38 [0.58 - 3.29] |
| ***Age of head of household at birth (years)*** |  |  |  |  |  |  |
| < 25 | 10/149 (6.7) | 2.67 [1.20 - 5.90] ** | 8/150 (5.3) | 0.77 [0.36 - 1.66] | 27/149 (18.1) | 0.97 [0.61 - 1.53] |
| 25 - 39 | 18/685 (2.6) | ref | 47/688 (6.8) | ref | 128/687 (18.6) | ref |
| 40 - 54 | 8/255 (3.1) | 1.20 [0.51 - 2.79] | 15/257 (5.8) | 0.84 [0.46 - 1.54] | 39/255 (15.3) | 0.79 [0.53 - 1.17] |
| 55 + | 7/230 (3.0) | 1.16 [0.48 - 2.82] | 17/231 (7.4) | 1.08 [0.61 - 1.93] | 50/229 (21.8) | 1.22 [0.84 - 1.76] |
| ***Infant season of birth*** |  |  |  |  |  |  |
| Warm & rainy (Jan – May) | 16/532 (3.0) | ref | 31/535 (5.8) | ref | 93/531 (17.5) | ref |
| Cool & dry (Jun – Sept) | 20/545 (3.7) | 1.23 [0.63 - 2.40] | 39/549 (7.1) | 1.24 [0.76 - 2.02] | 95/547 (17.4) | 0.99 [0.72 - 1.36] |
| Dry (Oct – Dec) | 7/242 (2.9) | 0.96 [0.39 - 2.37] | 17/242 (7.0) | 1.23 [0.67 - 2.27] | 56/242 (23.1) | 1.42 [0.98 - 2.06] * |
| **SOCIO-ECONOMIC STATUS** |  |  |  |  |  |  |
| ***Father's education level*** |  |  |  |  |  |  |
| Unknown | 2/93 (2.1) | 0.72 [0.16 - 3.21] | 3/93 (3.2) | 0.55 [0.16 - 1.85] | 14/93 (15.1) | 0.99 [0.54 - 1.84] |
| None or uncompleted primary | 12/285 (4.2) | 1.44 [0.67 - 3.12] | 25/287 (8.7) | 1.58 [0.91 - 2.76] | 68/284 (23.9) | 1.77 [1.23 - 2.54] ** |
| Completed primary | 14/434 (3.2) | 1.09 [0.52 - 2.29] | 30/436 (6.9) | 1.23 [0.72 - 2.08] | 85/434 (19.6) | 1.37 [0.97 - 1.92] * |
| Secondary or tertiary | 15/507 (3.0) | ref | 29/510 (5.7) | ref | 77/509 (15.1) | ref |
| ***Source of drinking water*** |  |  |  |  |  |  |
| Tap | 3/200 (1.5) | ref | 5/200 (2.5) | ref | 26/201 (12.9) | ref |
| Bore hole | 25/846 (2.9) | 2.00 [0.60 - 6.69] | 59/853 (6.9) | 2.90 [1.15 - 7.32] ** | 160/847 (18.9) | 1.57 [1.00 - 2.45] ** |
| River or lake | 15/268 (5.6) | 3.89 [1.11 - 13.64] ** | 23/268 (8.6) | 3.66 [1.37 - 9.81] ** | 57/267 (21.3) | 1.83 [1.10 - 3.03] ** |
| ***Dwelling category*** |  |  |  |  |  |  |
| 1 (best) | 2/197 (1.0) | ref | 6/197 (3.0) | ref | 34/198 (17.3) | ref |
| 2 | 5/172 (2.9) | 2.92 [0.56 - 15.24] | 10/173 (5.8) | 1.95 [0.69 - 5.49] | 26/172 (15.1) | 0.86 [0.49 - 1.50] |
| 3 | 12/412 (2.9) | 2.93 [0.65 - 13.20] | 28/416 (6.7) | 2.30 [0.94 - 5.64] * | 76/412 (18.4) | 1.09 [0.70 - 1.70] |
| 4 (worst) | 24/519 (4.6) | 4.72 [1.11 - 20.19] ** | 43/521 (8.2) | 2.86 [1.20 - 6.84] ** | 104/519 (20.0) | 1.21 [0.79 - 1.85] |
| Trend across categories |  | 1.51 [1.07 – 2.14] ** |  | 1.34 [1.06 – 1.68] ** |  | 1.09 [0.95 – 1.25] |
| ***Asset score (USD)*** |  |  |  |  |  |  |
| < 5 | 10/275 (3.6) | 3.05 [0.94 - 9.83] * | 20/277 (7.2) | 1.63 [0.82 - 3.25] | 60/275 (21.8) | 1.35 [0.90 - 2.03] |
| 5 - 9.99 | 12/261 (4.6) | 3.89 [1.24 - 12.21] ** | 20/262 (7.6) | 1.73 [0.87 - 3.45] | 49/261 (18.8) | 1.12 [0.73 - 1.71] |
| 10 - 49.99 | 17/456 (3.7) | 3.13 [1.04 - 9.38] ** | 32/458 (7.0) | 1.57 [0.84 - 2.95] | 79/457 (17.3) | 1.01 [0.69 – 1.47] |
| >= 50 | 4/327 (1.2) | ref | 15/329 (4.6) | ref | 56/327 (17.1) | ref |
| ***HH main source of income*** |  |  |  |  |  |  |
| Farming | 13/513 (2.5) | ref | 34/514 (6.6) | ref | 105/513 (20.5) | ref |
| Employment & letting | 6/209 (2.9) | 1.13 [0.43 - 3.03] | 12/210 (5.7) | 0.86 [0.43 - 1.69] | 28/209 (13.4) | 0.60 [0.38 - 0.94] ** |
| Piecework & gathering | 10/138 (7.2) | 3.00 [1.29 - 7.01] ** | 17/141 (12.0) | 1.93 [1.05 - 3.58] ** | 33/138 (23.9) | 1.22 [0.78 - 1.91] |
| Fishing | 1/133 (0.7) | 0.29 [0.04 - 2.25] | 3/134 (2.2) | 0.32 [0.10 - 1.07] * | 17/133 (12.8) | 0.57 [0.33 - 0.99] ** |
| Trade | 5/173 (2.9) | 1.14 [0.40 - 3.26] | 11/173 (6.3) | 0.96 [0.47 - 1.93] | 33/175 (18.9) | 0.90 [0.58 - 1.40] |
| Selling own goods and snacks | 3/60 (5.0) | 2.02 [0.56 - 7.32] | 4/61 (6.6) | 0.99 [0.34 - 2.89] | 11/59 (18.6) | 0.89 [0.45 - 1.77] |
| Other | 4/74 (5.4) | 2.20 [0.70 - 6.93] | 5/74 (6.8) | 1.02 [0.39 - 2.70] | 15/74 (20.3) | 0.99 [0.54 - 1.81] |
| **AGRICULTURE** |  |  |  |  |  |  |
| ***Growing maize*** |  |  |  |  |  |  |
| No | 7/205 (3.4) | ref | 19/205 (9.3) | ref | 44/205 (21.5) | ref |
| Yes | 36/1109 (3.2) | 0.95 [0.42 - 2.16] | 68/1116 (6.1) | 0.64 [0.37 – 1.08] * | 199/1110 (17.9) | 0.80 [0.55 - 1.15] |
| **HEALTH** |  |  |  |  |  |  |
| ***History of hospital admission*** |  |  |  |  |  |  |
| No | 3/1061 (0.3) | ref | 60/1066 (5.6) | ref | 194/1061 (18.3) | ref |
| Yes | 12/249 (4.8) | 1.68 [0.85 - 3.32] | 26/251 (10.4) | 1.94 [1.20 - 3.14] ** | 48/250 (19.2) | 1.06 [0.74 - 1.51] |
| ***History of traditional healer consultation*** |  |  |  |  |  |  |
| No | 28/928 (3.0) | ref | 45/934 (4.8) | ref | 166/929 (17.9) | ref |
| Yes | 15/373 (4.0) | 1.35 [0.71 - 2.55] | 42/374 (11.2) | 2.50 [1.61 - 3.88] *** | 77/373 (20.6) | 1.20 [0.88 - 1.61] |
| **NUTRITION & ANTHROPOMETRY** |  |  |  |  |  |  |
| ***Age introduction of water*** |  |  |  |  |  |  |
| < 4 months | 13/235 (5.5) | 2.07 [1.01 – 4.27] ** | 22/236 (9.3) | 1.64 [0.96 - 2.82] * | 46/234 (19.7) | 1.12 [0.77 - 1.64] |
| 4-5 months | 11/388 (2.8) | 1.03 [0.49 – 2.19] | 24/389 (6.2) | 1.05 [0.63 - 1.77] | 74/389 (19.0) | 1.08 [0.78 - 1.48] |
| ≥ 6 months | 19/692 (2.7) | ref | 41/697 (5.9) | ref | 124/693 (17.9) | ref |
| ***Age introduction of complementary food*** |  |  |  |  |  |  |
| < 4 months | 16/310 (5.2) | 1.97 [0.99 - 3.93] * | 28/310 (9.0) | 1.77 [1.06 - 2.95] ** | 62/309 (20.1) | 1.19 [0.85 - 1.68] |
| 4-5 months | 8/332 (2.4) | 0.90 [0.39 - 2.08] | 22/333 (6.6) | 1.26 [0.73 - 2.18] | 64/332 (19.3) | 1.13 [0.81 - 1.59] |
| ≥ 6 months | 18/671 (2.7) | ref | 36/677 (5.3) | ref | 117/672 (17.4) | ref |
| ***Age introduction of family food*** |  |  |  |  |  |  |
| < 6 months | 4/48 (8.3) | 2.80 [0.96 - 8.17] * | 5/48 (10.4) | 1.74 [0.67 - 4.52] | 12/48 (25.0) | 1.50 [0.77 - 2.92] |
| ≥ 6 months | 39/1240 (3.1) | ref | 78/1247 (6.2) | ref | 226/1240 (18.2) | ref |
| ***Wasting at baseline*** |  |  |  |  |  |  |
| No | 38/1182 (3.2) | ref | 67/1189 (5.6) | ref | 216/1189 (18.2) | ref |
| Moderate | 3/77 (3.9) | 1.23 [0.37 - 4.06] | 15/77 (19.5) | 4.07 [2.20 - 7.53] *** | 15/76 (19.7) | 1.11 [0.62 - 1.99] |
| Severe | 1/21 (4.8) | 1.51 [0.20 - 11.56] | 4/21 (19.0) | 3.96 [1.30 - 12.09] ** | 4/21 (19.0) | 1.06 [0.35 - 3.18] |
| ***Stunting at baseline*** |  |  |  |  |  |  |
| No | 33/1182 (2.8) | ref | 61/1189 (5.1) | ref | 33/1182 (2.8) | ref |
| Moderate | 7/88 (7.9) | 3.01 [1.29 - 7.01] ** | 16/88 (18.2) | 4.11 [2.26 - 7.49] *** | 32/87 (36.8) | 3.06 [1.92 - 4.87] *** |
| Severe | 3/25 (12.0) | 4.75 [1.35 - 16.65] ** | 10/25 (40.0) | 12.33 [5.32 - 28.57] *** | 17/25 (68.0) | 11.19 [4.76 - 26.29] *** |
| ***Underweight at baseline*** |  |  |  |  |  |  |
| No | 31/1227 (2.5) | ref | 55/1234 (4.5) | ref | 199/1229 (16.2) | ref |
| Moderate | 8/68 (11.8) | 5.14 [2.27 - 11.67] *** | 24/68 (35.3) | 11.69 [6.64 - 20.59] *** | 34/68 (50.0) | 5.18 [3.14 - 8.52] *** |
| Severe | 4/16 (25.0) | 12.86 [3.93 - 42.12]*** | 8/16 (50.0) | 21.44 [7.76 - 59.24] *** | 9/15 (60.0) | 7.76 [2.73 - 22.05] *** |
| ***Maternal malnutrition at follow-up*** |  |  |  |  |  |  |
| Yes | 1/43 (2.3) | 1.75 [0.23 - 13.42] | 6/86 (7.0) | 7.59 [2.78 - 20.76] *** | 6/241 (2.5) | 2.24 [0.83 - 6.03] |
| No | 17/1263 (1.3) | ref | 12/1227 (1.0) | ref | 12/1066 (1.1) | ref |

* p< 0.1 ; ** p< 0.05 ; *** p< 0.001
